# Gestational diabetes mellitus and interpregnancy weight change: A population-based cohort study

**DOI:** 10.1371/journal.pmed.1002367

**Published:** 2017-08-01

**Authors:** L. M. Sorbye, R. Skjaerven, K. Klungsoyr, N. H. Morken

**Affiliations:** 1 Department of Global Public Health and Primary Care, University of Bergen, Bergen, Norway; 2 Norwegian National Advisory Unit on Women’s Health, Oslo University Hospital, Rikshospitalet, Oslo, Norway; 3 Medical Birth Registry of Norway, Norwegian Institute of Public Health, Bergen, Norway; 4 Department of Clinical Science, University of Bergen, Bergen, Norway; Chinese University of Hong Kong, CHINA

## Abstract

**Background:**

Being overweight is an important risk factor for Gestational Diabetes Mellitus (GDM), but the underlying mechanisms are not understood. Weight change between pregnancies has been suggested to be an independent mechanism behind GDM. We assessed the risk for GDM in second pregnancy by change in Body Mass Index (BMI) from first to second pregnancy and whether BMI and gestational weight gain modified the risk.

**Methods and findings:**

In this observational cohort, we included 24,198 mothers and their 2 first pregnancies in data from the Medical Birth Registry of Norway (2006–2014). Weight change, defined as prepregnant BMI in second pregnancy minus prepregnant BMI in first pregnancy, was divided into 6 categories by units BMI (kilo/square meter). Relative risk (RR) estimates were obtained by general linear models for the binary family and adjusted for maternal age at second delivery, country of birth, education, smoking in pregnancy, interpregnancy interval, and year of second birth. Analyses were stratified by BMI (first pregnancy) and gestational weight gain (second pregnancy). Compared to women with stable BMI (−1 to 1), women who gained weight between pregnancies had higher risk of GDM—gaining 1 to 2 units: adjusted RR 2.0 (95% CI 1.5 to 2.7), 2 to 4 units: RR 2.6 (2.0 to 3.5), and ≥4 units: RR 5.4 (4.0 to 7.4). Risk increased significantly both for women with BMI below and above 25 at first pregnancy, although it increased more for the former group. A limitation in our study was the limited data on BMI in 2 pregnancies.

**Conclusions:**

The risk of GDM increased with increasing weight gain from first to second pregnancy, and more strongly among women with BMI < 25 in first pregnancy. Our results suggest weight change as a metabolic mechanism behind the increased risk of GDM, thus weight change should be acknowledged as an independent factor for screening GDM in clinical guidelines. Promoting healthy weight from preconception through the postpartum period should be a target.

## Abstract 2

Linn Sorbye and colleagues identify weight gain between pregnancies as an independent risk factor for gestational diabetes, using data from a large Norwegian birth registry.

## Author summary

**Why was this study done?:**

- Being overweight during pregnancy is an important risk factor for Gestational Diabetes Mellitus (GDM); however, the underlying mechanisms are not clear.
- Recent evidence has found that women who increase their weight from first to second pregnancy increase the risk of GDM, suggesting weight as a causal mechanism behind GDM.
- Research is not consistent on whether the association is dependent on the woman’s weight status when she enters her first pregnancy.

**What did the researchers do and find?:**

- In this observational cohort study, we used data from the population-based Medical Birth Registry of Norway and included 24,198 mothers with their first and second pregnancy during 2006–2014.
- We investigated if a change in Body Mass Index (BMI) between first and second pregnancy affected the risk of GDM in the second pregnancy and if the association was dependent on the woman’s prepregnant BMI in first pregnancy
- The risk of GDM in second pregnancy increased with increasing weight gain between pregnancies, and more strongly among women who had a BMI below 25 (kilo/meters squared) in first pregnancy.
- Decreasing BMI by >2 units from first to second pregnancy had a preventive effect on GDM in overweight and obese women.

**What do these findings mean?:**

- Weight gain between pregnancies should be evaluated as an independent risk factor for screening GDM in clinical antenatal guidelines.
- Our results need to be replicated in other populations, and they underline the importance of future research on pathophysiologic mechanisms behind the development of GDM.
- Efforts to promote healthy weight in the reproductive population need to expand their focus to include healthy maternal weight from preconception throughout reproduction.

## Introduction

Worldwide, overweight has reached epidemic proportions [1, 2], with serious consequences for reproductive health. Being overweight during pregnancy increases the risk of complications in pregnancy, in childbirth, and for the newborn child [3–7] and is an important risk factor for Gestational Diabetes Mellitus (GDM) [8–11]. GDM is defined as glucose intolerance of various degrees that is first detected during pregnancy [12], and the prevalence of GDM has increased, with variation by countries [11, 13–15]. GDM increases the risk of immediate adverse pregnancy and infant outcomes [16] and, in the long term, the risk of metabolic syndrome and Type 2 Diabetes Mellitus in the mothers [17–19]. Children born to GDM mothers have an increased risk of high birthweight and being overweight in adolescence [20].

The underlying genetic, physiological, and environmental factors behind the development of GDM are not fully understood [12, 21]. Both prepregnant Body Mass Index (BMI) and gestational weight gain are risk factors for GDM, but evidence of an independent or joint effect is inconsistent [22–24]. Most observational studies have not been able to evaluate shared genetic and early environmental risk factors within families. Recent studies have suggested interpregnancy weight change as part of the causal mechanism behind the risk of GDM and other adverse pregnancy outcomes in the second pregnancy; however, knowledge of this is scarce [25–27]. It has also been suggested that the effect of interpregnancy weight change may depend on the woman’s prepregnant BMI during her first pregnancy [25–27]. One study found that women gaining weight between their first and second pregnancy increased their risk for GDM in their second pregnancy, even though they were normal weight during both pregnancies [25]. Previous studies have not been able to study the importance of gestational weight gain upon this association. We estimated the association between prepregnancy BMI changes from the first to the second pregnancy and GDM in the second pregnancy for women giving birth in Norway. Secondly, we explored the roles of prepregnant BMI in first pregnancy and gestational weight gain in second pregnancy as effect modifiers.

## Methods

### Data sources

This observational cohort study complies with the guidelines of the Declaration of Helsinki. The project was approved by the Regional Ethics Committee, REK VEST (2015/1728). Informed consent was not required as data were de-identified, and the researchers did not have any additional patient contact. We used prospectively collected data from the population-based Medical Birth Registry of Norway (MBRN) [28]. Using the unique national identification number, each child born was linked to his/her mother, so that each record consisted of the mother and her successive 2 first births (2006–2014). The MBRN was established in 1967 and is based on compulsory notification of all live- and stillbirths from 16 weeks of gestation (12 weeks from 2002). Midwives and doctors attending the birth complete a standardized notification form on demographics, maternal health before and during pregnancy, previous reproductive history, complications during pregnancy and delivery, and pregnancy outcomes [29]. Data on maternal height and weight prior to conception are self-reported. Weight at the end of pregnancy is defined as maternal weight measured at last antenatal visit or when arriving at the delivery ward. The collection of maternal height and weight information in MBRN began in 2006 when Norway adopted a revised electronic birth notification system, but the implementation of the system by all delivery units was not complete until 2014. The proportion of registered maternal height and weight in MBRN steadily increased from 0.1% in 2006 to 71.6% of births in 2014 [S1 Fig]. Complete data on prepregnancy height and weight is available for 24% of the women having their first 2 deliveries during 2006–2014. During this period, the distribution of women across the different BMI categories has been stable over time, suggesting that even in years with low reporting, the data are likely representative for the population [30]. Data on country of birth (Nordic defined as Norway, Sweden, Denmark, Finland, and Iceland) was obtained from The National Population Registry, Statistics Norway. Maternal education, defined as the highest achieved years of education classified according to The Norwegian Standard Classification of Education, was obtained from the National Education Database, Statistics Norway.

### Inclusions and definitions

**Fig 1:**
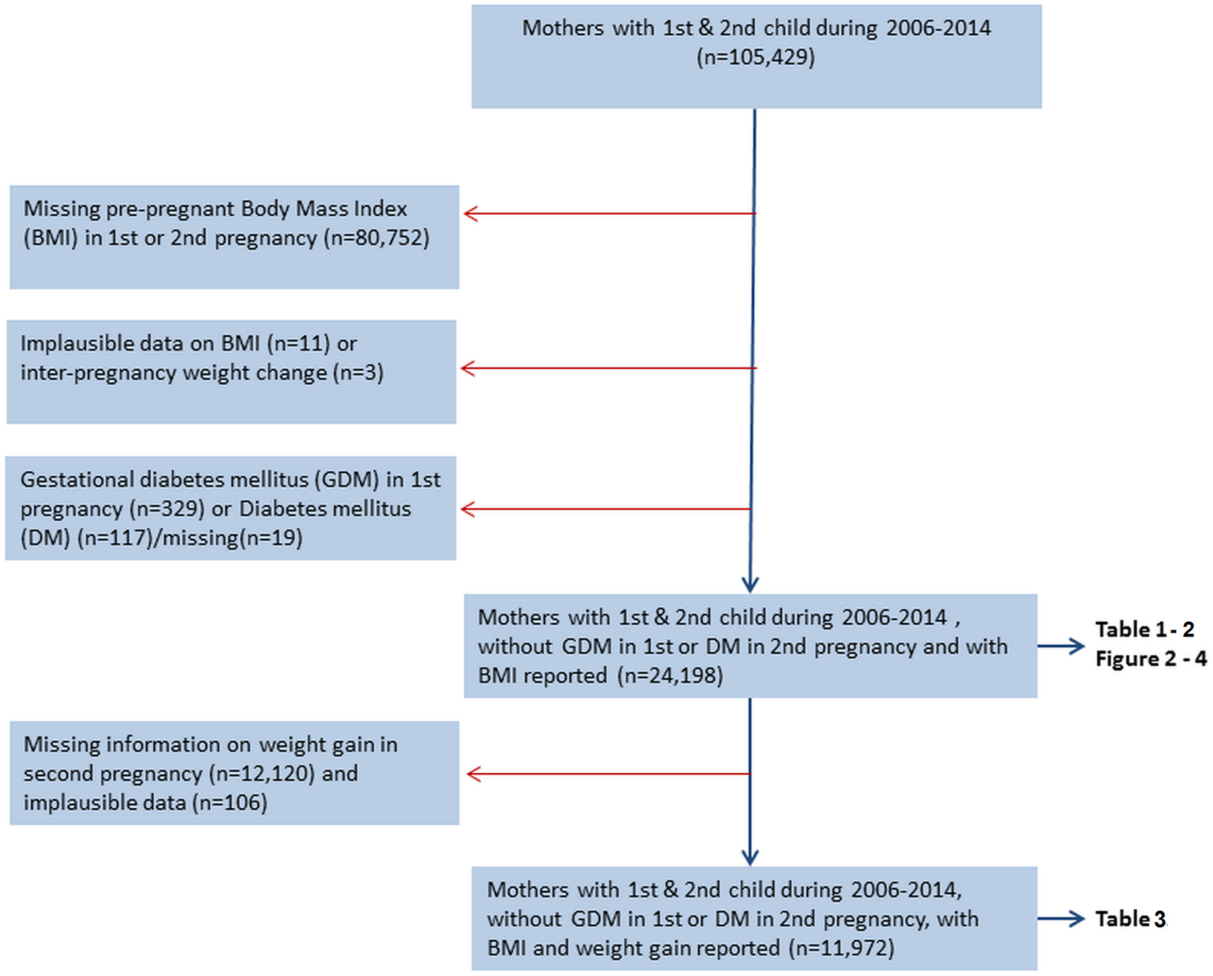
Flow chart showing inclusion and exclusions, The Medical Birth Registry of Norway, 2006–2014.

Fig 1 shows a flow chart of the included and excluded women in the study.

Prepregnant BMI was calculated from weight in kilograms (kg) divided by height in meters squared (m^2^). Our main exposure was interpregnancy weight change defined as prepregnant BMI in second pregnancy minus prepregnant BMI in first pregnancy. Gestational weight gain in second pregnancy was calculated as weight (kg) at the end of pregnancy minus prepregnant weight. A total of 105,429 women delivered their first 2 births during 2006–2014. To be included, women had to have prepregnancy height and weight reported for both pregnancies (*n* = 24,677). We excluded women with prepregnant BMI below 15 (*n* = 11) and interpregnancy weight change above +30 and below −30 BMI units (kg/m^2^) (*n* = 3), as they were considered implausible. To evaluate first-time occurrence of GDM in the second pregnancy, women diagnosed with GDM in their first pregnancy were excluded (*n* = 329). In the analyses exploring the potential effect modifying role of gestational weight gain in second pregnancy, only women who had their weight registered both before and at the end of their second pregnancy were included in the analysis (*n* = 11,972). A weight gain above 70 kg during second pregnancy was considered implausible (*n* = 6)

We categorized prepregnancy BMI into underweight (<18.5), normal weight (18.5–24.9), overweight (25–29.9), and obese (≥30) [31]. Prepregnant BMI in first pregnancy was further dichotomized into <25 kg/m^2^ versus ≥25 kg/m^2^ for stratified analyses. We categorized interpregnancy weight change into 6 groups: <−2, −2 to <−1, −1 to <1 (reference), 1 to <2, 2 to <4, and ≥4 kg/m^2^[26, 27]. Gestational weight gain was grouped into the following: weight loss, 0–7.9 kg, 8–15.9 kg, and ≥16 kg [32]. In stratified analyses, women with a weight loss were excluded, and gestational weight gain was dichotomized by the median value: 0–13.9 kg and ≥14 kg. The main outcome was GDM (yes/no), notified by a check box on the birth notification form or as free text coded at the MBRN according to the International Classification of Diseases (ICD) version 10. The diagnostic criteria and screening strategy for GDM are given in the national guidelines for antenatal care and obstetrics made by the Norwegian Society of Gynecology and Obstetrics (in 1998, 2008, and 2014) [33]. GDM is diagnosed when fasting plasma glucose levels are <7.0 millimole (mmol)/liter (l) and serum blood glucose following an oral glucose tolerance test (OGTT) (2 hours after intake of 75 grams oral glucose) is ≥7.8 mmol/l but <11.1 mmol/l. Women follow a standardized antenatal program with regular visits, which include urine stix testing for glucose at each visit. Independent of gestational week, an OGTT is recommended when the stix is positive (≥+++). Women with risk factors (age > 38 years, close relatives with Gestational Diabetes type 1/2, immigrants from the Indian Subcontinent or North Africa, prepregnant BMI > 27 kg/m^2^, or prior GDM) will be offered an OGTT as early as possible in pregnancy, and if negative, it will be repeated in week 28–30 [33].

Maternal smoking habits were reported at the end of pregnancy, and women could answer “no,” “occasionally,” “daily,” or they could decline to answer (*n* = 2,592). Daily and occasional smokers (*n* = 110) were considered smokers. Interpregnancy interval was calculated as the date of the second birth minus the date of first birth minus the pregnancy length of the second pregnancy, and it was categorized into <12, 12 to <24 (reference), 24 to <36, and ≥36 months. In stratified analyses, the interpregnancy interval was dichotomized: <24 and ≥24 months. The gestational age of the second pregnancy was based on second trimester ultrasound estimations or, if missing, on the mother’s last menstrual period.

### Statistics

Chi-square tests were used to investigate associations and linear trends between variables. General linear models with extension for the binary family were used to estimate the association between interpregnancy weight change from the first to second pregnancy and the risk of GDM in the second pregnancy. Relative risks (RRs) with 95% CI were calculated for interpregnancy weight-change categories. Maternal age at second delivery (<25, 25–29, 30–34, ≥35 years), country of birth (Nordic/non-Nordic), highest achieved years of education (<11, 11–14, ≥14 years), smoking during pregnancy (yes/no), interpregnancy interval (<12, 12 to <24, 24 to <36, ≥36 months), and year of the second birth (continuous variable) were considered as possible confounders [34] and were adjusted for in the multivariable binary regression model. The presented adjusted models (*n* = 20,824) had missing information on covariates in 3,374 (13.9%) women. Missing values for smoking, maternal education, and maternal country of birth were therefore handled by missing imputation using chained equations (MICE) with logistic regression for smoking and maternal country of birth and multinomial logistic regression for maternal education [35] [S1 Table]. When evaluating a potential effect modification by prepregnant BMI in the first pregnancy (BMI < 25 and BMI ≥ 25), and gestational weight gain (<14 and ≥14) in the second pregnancy, we included an interaction term in the multiplicative model evaluated by Likelihood ratio test. To compare the risk of GDM in women with BMI ≥ 25 and BMI < 25 in the first pregnancy, we included interpregnancy BMI change as a continuous variable in the interaction term (Likelihood ratio test, Poisson regression). Associations were considered statistically significant at the 5% level. All statistical analyses were performed using STATA IC Statistical software version 14 and Statistical Package for the Social Sciences, version 23 (www.spss.com).

## Results

A total of 24,198 mothers were included in the main analysis. Of these, 12,078 (50%) women had information on gestational weight gain (GWG) in their second pregnancy and were included in the analysis evaluating effect modification by GWG. Population characteristics by GDM in second pregnancy are shown in Table 1.

**Table 1:** Population characteristics according to GDM in second pregnancy. A population-based cohort study (*n* = 24,198), The Medical Birth Registry of Norway, 2006–2014.

	GDM in Second Pregnancy			
	Yes	%	Total	*p*-value*
**BMI in first pregnancy** (kg/m^2^)				
<18.5	13	1.2	1,065	<0.001
18.5–24.9	169	1.1	16,052	
25–29.9	142	3.0	4,812	
≥30.0	115	5.1	2,269	
**BMI in second pregnancy** (kg/m^2^)				<0.001
<18.5	5	0.6	897	
18.5–24.9	120	0.8	14,812	
25–29.9	137	2.5	5,534	
≥30.0	177	6.0	2,955	
**Gestational weight gain in second pregnancy** (kg)				<0.001
<−0.1	4	4.0	100	
0–7.9	57	4.5	1,274	
8–15.9	104	1.6	6,489	
≥16.0	50	1.2	4,215	
Missing	224	1.8	12,120	
**Maternal age** (years)				<0.001
<25	32	1.1	3,014	
25–29	142	1.7	8,476	
30–34	151	1.7	8,987	
≥35	114	3.1	3,721	
**Maternal country of birth**				<0.001**
Nordic	315	1.6	19,828	
Non-Nordic	121	2.9	4,215	
Missing	3	1.9	155	
**Maternal education** (years)				0.034
<11	73	2.1	3,460	
11–13	124	2.0	6,323	
≥14	225	1.6	13,678	
Missing	17	2.3	737	
**Smoking**				0.516**
No	370	1.8	20,616	
Yes	15	1.5	990	
Missing	54	2.1	2,592	
**Interpregnancy interval** (months)				<0.001
<12	70	1.5	4,806	
12 to <24	151	1.5	10,342	
24 to <36	114	2.0	5,811	
≥36	104	3.2	3,210	
Missing	0	0.0	29	

*Chi-square test for linear trend.

**Pearson Chi square.

BMI, Body Mass Index; GDM, Gestational Diabetes Mellitus

The overall absolute risk of GDM in second pregnancy was 18.1 per 1,000 pregnancies (439/24,198), and the prevalence of GDM increased with increasing level of prepregnant BMI in second pregnancy (*p* for trend <0.001). Interpregnancy weight change from the first to second pregnancy was relatively stable (−1 to <1 BMI units) in 47.6% of women (*n* = 11,512), while 16.8% (*n* = 4,076) of women had a weight loss of >1 BMI unit and 35.6% (*n* = 8,610) of women gained weight ≥1 BMI unit. To evaluate the representativeness of our study population, we compared the study population with the general population in MBRN during 2006–2014 [S10 Table].

During the second pregnancy, 0.8% (100/12,078) of women lost weight (<0 kg), 10.5% had a GWG of 0 kg to 7.9 kg, 53.7% had a GWG of 8 kg to 15.9 kg, and 34.9% had a GWG of ≥16 kg. Overall, we found a negative correlation between interpregnancy weight change (kg/m^2^) and gestational weight gain (kg) in second pregnancy (r −0.20, *p* < 0.001, *n* = 12,078). For each BMI unit increase from the first to second pregnancy, gestational weight gain in the second pregnancy decreased by 0.53 kg (β: −0.53, 95% CI: −0.58 to −0.49).

### Interpregnancy weight change and GDM in second pregnancy

**Fig 2:**
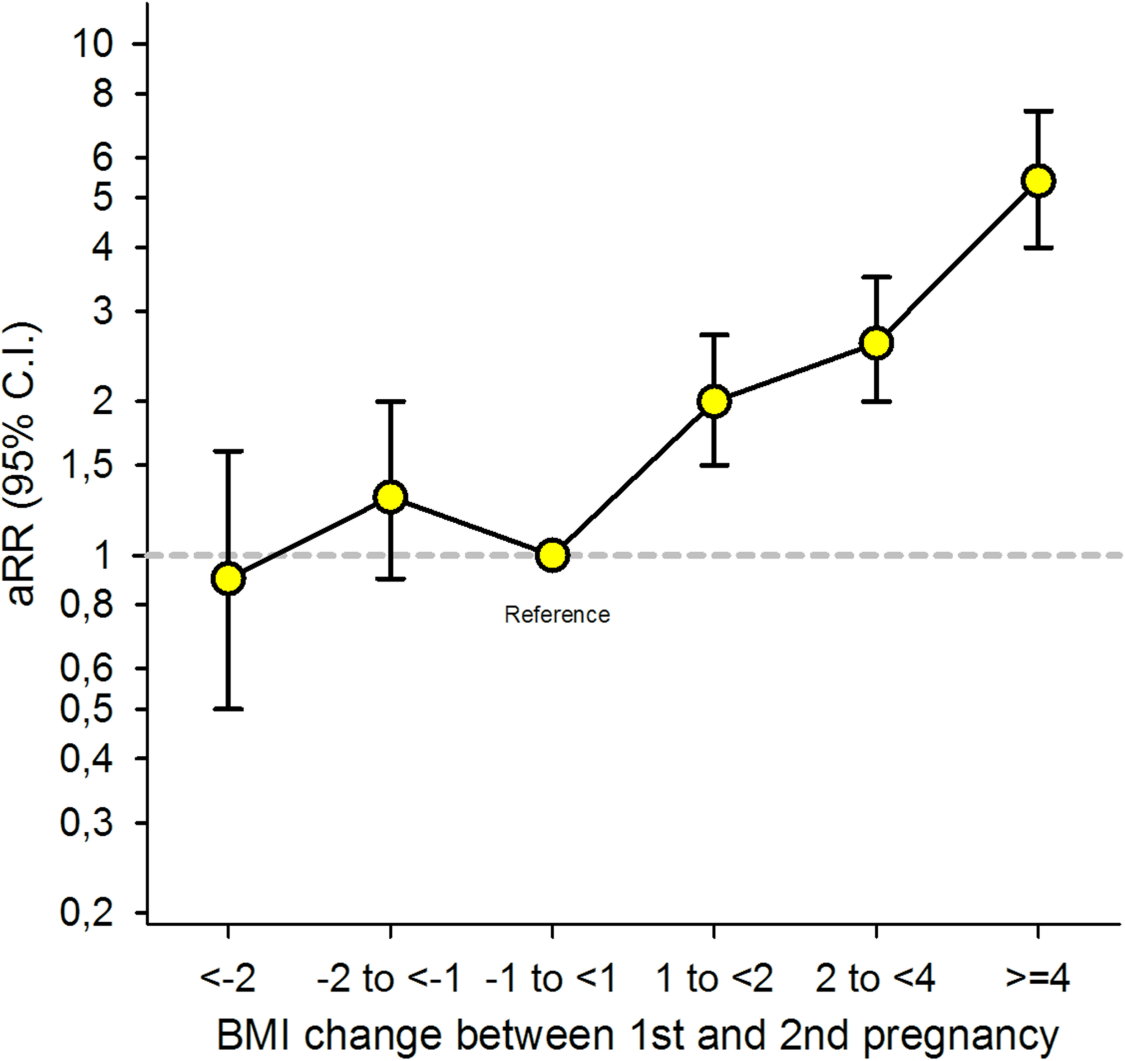
Overall adjusted (a) relative risk (RR) for Gestational Diabetes Mellitus (GDM) in second pregnancy by change in Body Mass Index (BMI) between the first and second pregnancy (*n* = 24,198), The Medical Birth Registry of Norway 2006–2014. Analysis adjusted for maternal age during the second pregnancy (<25 [reference], 25–29, 30–34, ≥35 years), maternal country of birth (Nordic [reference]/non-Nordic), maternal education (<11, 11–13, ≥14 [reference] years), smoking during pregnancy (no [reference],/yes), interpregnancy interval (<12, 12–23 [reference], 24–35, ≥36 months), and year of second birth (continuous).

Overall, the risk of GDM in the second pregnancy increased with increasing interpregnancy weight gain between the first and second pregnancy [Fig 2] [Table 2].

**Table 2:** Overall risk for GDM in second pregnancy by interpregnancy change in BMI (*n* = 24,198), The Medical Birth Registry of Norway 2006–2014.

Interpregnancy BMI Change (kg/m^2^)			RR for GDM in Second Pregnancy			
	Total	GDM /1000	Crude RR	95% CI	aRR*	95% CI
**<−2**	15/1,692	8.9	0.8	0.5–1.4	0.9	0.5–1.6
**−2 til <−1**	34/2,384	14.3	1.3	0.9–1.9	1.3	0.9–2.0
**−1 til <1**	126/11,512	10.9	1.0	Reference	1.0	Reference
**1 til <2**	79/3,814	20.7	1.9	1.4–2.5	2.0	1.5–2.7
**2 til <4**	97/3,279	29.6	2.7	2.1–3.5	2.6	2.0–3.5
**≥4**	88/1,517	58.0	5.3	4.1–6.9	5.4	4.0–7.4
**Total**	439/24,198	18.1	24,198		20,824	

a, adjusted; BMI, Body Mass Index; GDM, Gestational Diabetes Mellitus; RR, relative risk.

*Analysis adjusted for maternal age during the second pregnancy (<25 (reference), 25–29, 30–34, ≥35 years), maternal country of birth (Nordic [reference]/non-Nordic), maternal education (<11, 11–13, ≥14 [reference] years), smoking in pregnancy (no[reference],/yes), interpregnancy interval (<12, 12–23 [reference], 24–35, ≥36 months), and year of the second birth (continuous).

Women who gained between 1 and 2 BMI units (kg/m^2^) had a doubled risk (adjusted [a] RR 2.0, 95% CI: 1.5–2.7), women gaining between 2 and 4 units had a 2.6x risk (a RR 2.6, 95% CI: 2.0–3.5), and women gaining ≥4 BMI units had a 5-fold risk of GDM in second pregnancy (a RR 5.4, 95% CI: 4.0–7.4) compared to women with stable interpregnancy weight (–1 to <1). The adjusted model with and without missing imputation can be seen in [S1 Table].

### Prepregnant BMI in first pregnancy, interpregnancy weight change, and GDM

**Fig 3:**
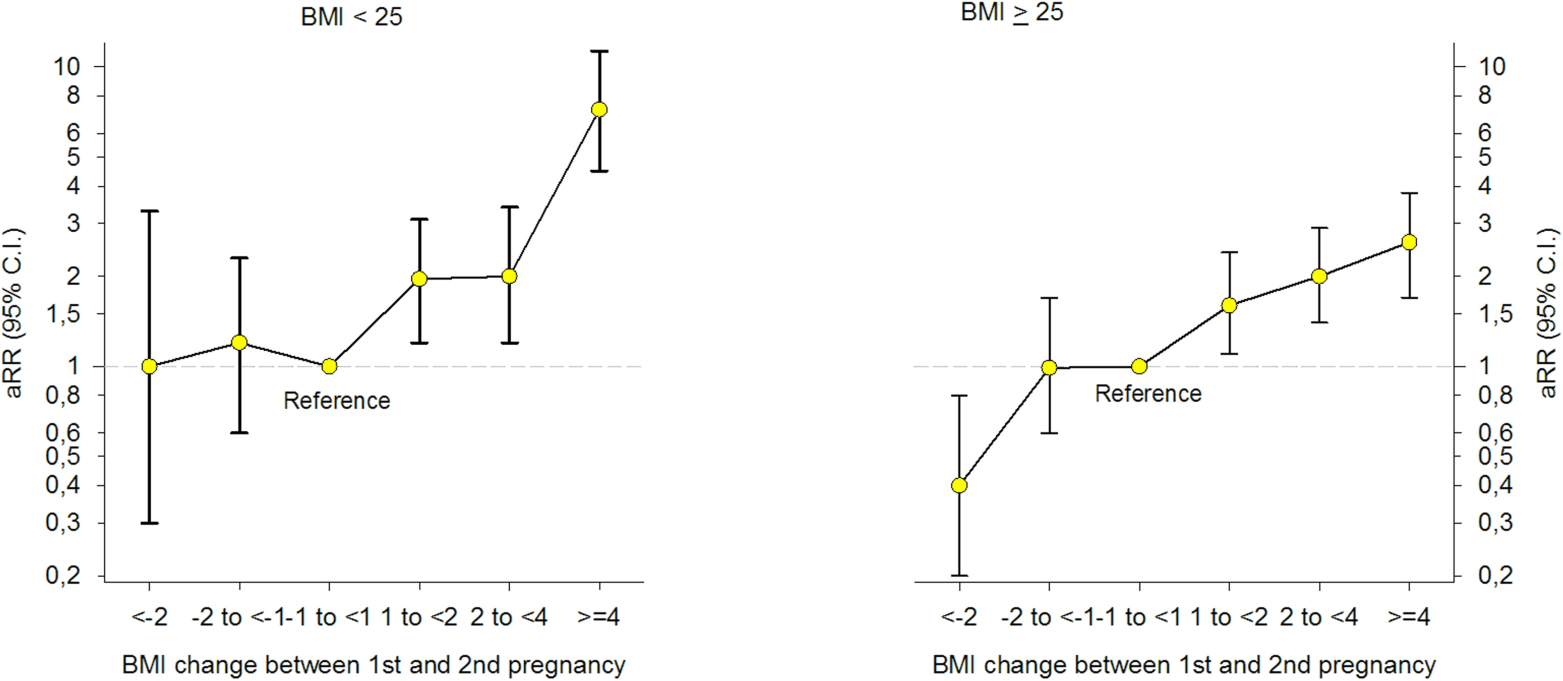
Adjusted (a) relative risk (RR) for Gestational Diabetes Mellitus (GDM) by change in Body Mass Index (BMI) between first and second pregnancy, stratified by BMI < 25 and BMI ≥ 25 in first pregnancy (*n* = 24,198), The Medical Birth Registry of Norway 2006–2014. Analyses adjusted for maternal age in the second pregnancy (<25 [reference], 25–29, 30–34, ≥35 years), maternal country of birth (Nordic [reference]/non-Nordic), maternal education (<11, 11–13, ≥14 [reference] years), smoking during pregnancy (no [reference]/yes), interpregnancy interval (<12, 12–23 [reference], 24–35, ≥36 months), and year of the second birth (continuous).

In stratified analyses by prepregnant BMI in the first pregnancy, the risk estimates for GDM increased with increasing weight gain between pregnancies and were stronger in women with BMI < 25 in the first pregnancy [Fig 3] [S2 Table]. Women who were overweight (BMI ≥ 25) in their first pregnancy and who reduced their BMI by ≥2 units prior to their second pregnancy, had a 60% lower risk of GDM in the second pregnancy [a RR 0.4, 95% CI: 0.2–0.8].

Results from the different adjusted models with and without missing imputation are seen in [S3 Table]. When excluding second pregnancies with gestational age below 37 weeks, multiple pregnancies and hypertensive disorders (hypertension during pregnancy, preeclampsia, eclampsia, and Hemolysis Elevated Liver enzymes and Low Platelet counts [HELLP]) (*n* = 1,755), results remained unchanged [S4 Table]. The prevalence of GDM in second pregnancy was significantly higher in non-Nordic compared to Nordic women (2.9% versus 1.6%, *p* < 0.001) [Table 1]. When we stratified the analysis by the mother’s country of birth [S5 Table] and maternal age [S6 Table], the association between interpregnancy weight change and GDM in the second pregnancy remained unchanged. Stratifying the analyses by interpregnancy interval (<24 and ≥24 months) revealed a stronger association between interpregnancy weight gain and GDM in women with an interval below 24 months [S7 Table] (*p* = 0.001 for interaction, Likelihood-ratio test, *n* = 20,824).

**Fig 4:**
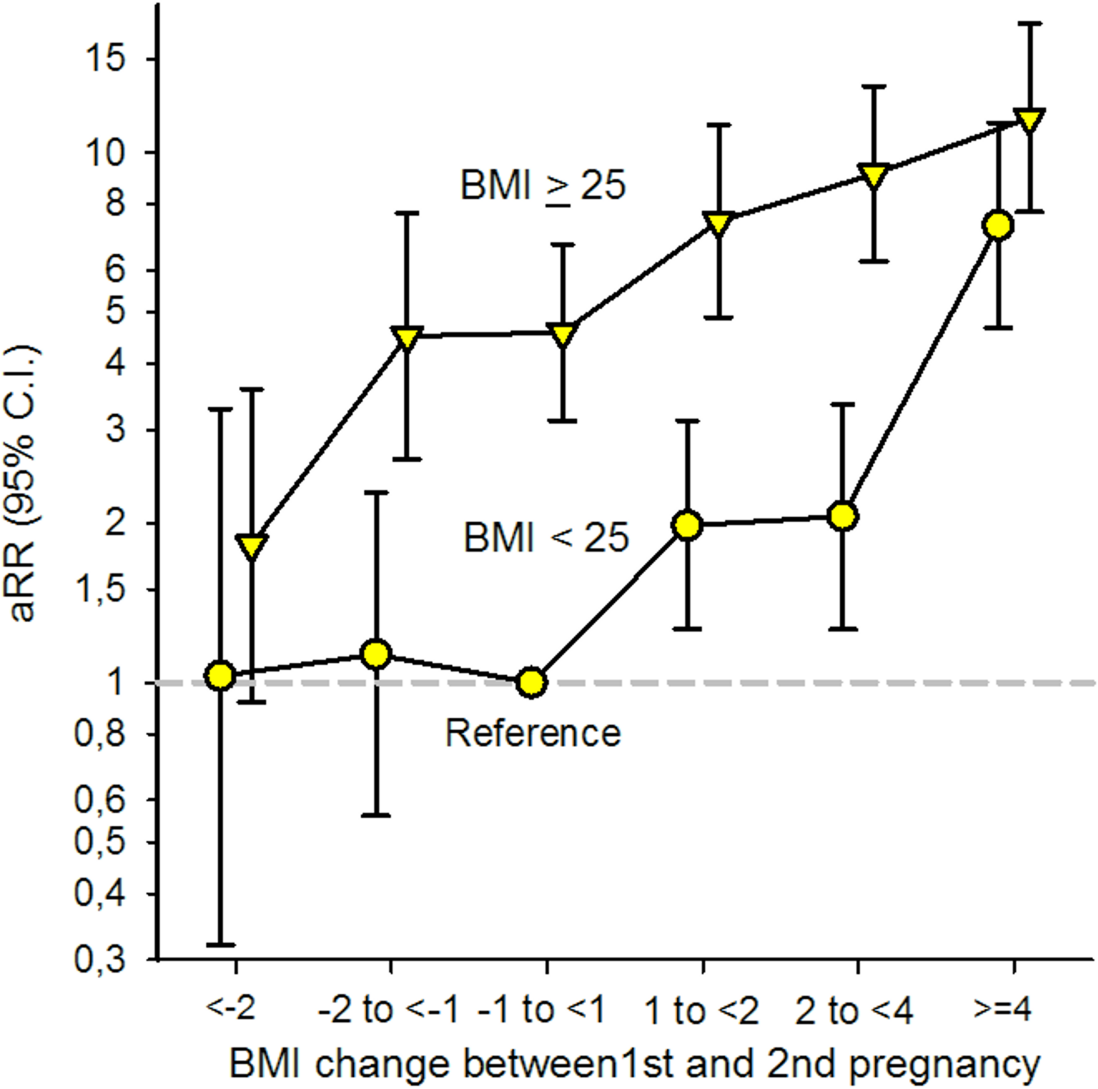
Adjusted (a) relative risk (RR) for Gestational Diabetes Mellitus (GDM) by change in Body Mass Index (BMI) between first and second pregnancy and BMI < 25 and BMI ≥ 25 in first pregnancy (*n* = 24,198), The Medical Birth Registry of Norway 2006–2014. Analysis adjusted for maternal age in second pregnancy (<25 [reference], 25–29, 30–34, ≥35 years), maternal country of birth (Nordic [reference]/non-Nordic), maternal education (<11, 11–13, ≥14 [reference] years), smoking during pregnancy (no [reference]/yes), interpregnancy interval (<12, 12–23 [reference], 24–35, ≥36 months), and year of second birth (continuous).

There was a significant interaction between prepregnant BMI (<25 and ≥25) in the first pregnancy and interpregnancy weight change (*p* = 0.009, Likelihood-ratio test). To test if the association between interpregnancy weight gain and GDM was stronger in women with BMI < 25 in their first pregnancy, we excluded women with interpregnancy weight change <−1 unit (*n* = 4,076) and added interpregnancy weight change as a continuous variable in the interaction term. Likelihood-ratio test confirmed the heterogeneity in risk (*p* < 0.001, Poisson regression analysis). To compare the risk of GDM in the second pregnancy in women with prepregnant BMI in their first pregnancy below or above 25 in 1 model, we included both groups in the same model using women with stable BMI (−1 to <1 BMI unit) and a prepregnant BMI < 25 in their first pregnancy as the common reference category. Having BMI ≥ 25 in the first pregnancy was associated with a higher risk of GDM across all interpregnancy weight change categories when compared to women in the reference group [Fig 4].

### The role of prepregnant BMI in second pregnancy

We defined prepregnant BMI in the second pregnancy as part of the exposure and the effect that we wished to study; therefore, we did not adjust for BMI in the second pregnancy. In order to find out if the association between interpregnancy weight change and GDM was independent of BMI in second pregnancy, we analyzed women who had prepregnant BMI below 25 both in their first and second pregnancy (*n* = 14,857) and found the same trend [S8 Table]. In this population, 0.8% (*n* = 118) developed GDM during the second pregnancy. When stratifying the analyses according to BMI in second pregnancy (*n* = 24,198), the association between interpregnancy weight gain and GDM remained [S9 Table].

### Gestational weight gain in second pregnancy, interpregnancy weight change, and GDM

Overall, we found an inverse linear association between gestational weight gain and GDM in second pregnancy (*p* for trend <0.001). After excluding women with negative gestational weight gain, the crude RR of GDM in the second pregnancy of women with gestational weight gain 0–13.9 kg was 2.0 (95% CI: 1.5–2.7), compared to women with ≥14 kg (*n* = 11,972). When we adjusted for prepregnant BMI in second pregnancy and confounders in our model, the RR of GDM in women with gestational weight gain 0–13.9 kg compared to women with ≥14 kg, was 1.9 (95% CI: 1.4–2.5). Testing for an interaction between gestational weight gain (0–13.9 and ≥14 kg) in the second pregnancy and interpregnancy weight change in a binary regression model gave a nonsignificant interaction term (*p* = 0.822, Likelihood-ratio test, *n* = 9,978). When we assessed the risk of GDM by interpregnancy weight change in strata of gestational weight gain in second pregnancy, we confirmed the positive association in both groups. The strongest association was found in women gaining ≥14 kg in their second pregnancy, although estimates were not significantly different [Table 3].

**Table 3:** RR for GDM in second pregnancy by interpregnancy change in BMI, stratified by GWG in second pregnancy (*n* = 11,972), The Medical Birth Registry of Norway 2006–2014.

BMI Change Units kg/m2	GWG in second pregnancy 0–13.9 kg						GWG in second pregnancy ≥14.0 kg					
	N	GDM /1000	CrudeRR	95% CI	a RR*	95% CI	N	GDM /1000	Crude RR	95% CI	a RR*	95% CI
**<−2**	3/281	10.7	0.7	0.2–2.2	0.6	0.1–2.4	3/504	6.0	0.8	0.2–2.8	0.6	0.2–2.8
**−2 to <−1**	7/494	14.2	0.9	0.4–2.0	0.8	0.3–1.9	6/679	8.8	1.2	0.5–3.0	0.9	0.3–2.8
**−1 to <1**	40/2,561	15.6	1.0	Reference	1.0	Reference	22/3,059	7.2	1.0	Reference	1.0	Reference
**1 to <2**	18/994	18.1	1.2	0.7–2.0	1.1	0.6–1.9	15/981	15.3	2.1	1.1–4.1	2.1	1.0–4.2
**2 to <4**	38/967	39.3	2.5	1.6–3.9	2.2	1.4–3.6	15/695	21.6	3.0	1.6–5.8	2.8	1.4–5.8
**≥4**	32/508	63.0	4.0	2.6–6.4	4.2	2.5–6.9	11/249	44.2	6.1	3.0–12.5	4.4	1.8–10.4
**Total**	138/5,805	23.8			4,808		72/6,167	11.7			5,170	

*Adjusted for maternal age in second pregnancy (<25 (reference), 25–29, 30–34, ≥35 years), maternal country of birth (Nordic [reference]/Non-Nordic), maternal education (<11, 11–13, ≥14 [reference] years), smoking during pregnancy (no [reference]/yes), interpregnancy interval (<12, 12–23 [reference], 24–35, ≥36 months), and year of second birth (continuous).

a, adjusted; BMI, Body Mass Index; GDM, Gestational Diabetes Mellitus; GWG, gestational weight gain

## Discussion

### Principal findings

We found a higher risk of GDM in women who increased their weight by ≥1 BMI unit from their first to second pregnancy compared to women with stable weight (−1 to <1 unit change). The risk estimates for GDM increased with increasing weight gain and applied to both normal-weight (prepregnant BMI < 25) and overweight (prepregnant BMI ≥ 25) women in their first pregnancy. Women with a BMI < 25 in their first pregnancy had the strongest association between interpregnancy weight gain and GDM. A preventive effect on GDM was seen in overweight women (first pregnancy) who reduced their weight by ≥2 BMI units until the second pregnancy.

### Interpregnancy weight change, BMI in first pregnancy, and the risk of GDM

Previous studies on interpregnancy weight change and risk of GDM are generally consistent with our findings with some exceptions [25, 36–39]. A Swedish population-based study found that the risk for GDM in the second pregnancy increased linearly with interpregnancy weight gain and was also present in women whose BMI was <25 in both pregnancies [25], consistent with our results. However, for overweight and obese women (BMI > 25 in first pregnancy), they found that only those with a large interpregnancy weight gain of ≥3 BMI units had an increased risk of GDM [25]. A regional cohort study from Belgium found an increased risk of GDM in women who had an interpregnancy weight gain of ≥1 BMI unit but only among women with BMI < 25 in their first pregnancy[37]. An American study also found, as we did, that the risk of GDM associated with interpregnancy weight gain applied both to women with BMI below and above 25 in first pregnancy. The risk estimates were also higher in women who had BMI < 25 in their first pregnancy, similar to our results [36].

We found a preventive effect of losing weight ≥2 BMI units from first to second pregnancy in women who had BMI ≥ 25 in first pregnancy, when compared to women with stable BMI in this group. Some studies have confirmed this [36, 38, 39], while others have not found any preventive effect of reducing weight on the risk of GDM [25, 37].

### Gestational weight gain and GDM

Obese women tend to gain less weight during pregnancy and also have the highest baseline risk of GDM; therefore, it is important to take BMI into consideration when examining gestational weight gain and GDM. We found the highest risk of GDM in women who had the lowest gestational weight gain, which has been confirmed in other studies [22, 37]. Reverse causation is a possible explanation, as women with GDM are closely monitored and receive advice on nutrition and physical activity, which may lead to lower gestational weight gain during the second pregnancy. Few studies have included gestational weight gain during second pregnancy when exploring the association between interpregnancy weight change and GDM. A study from Belgium found that inadequate gestational weight gain in second pregnancy was associated with GDM both in women who were normal weight or overweight in their first pregnancy [37].

### Pathophysiological mechanisms behind GDM

GDM is characterized by a decreased insulin sensitivity together with an inadequate insulin response [12]. Obesity is an important risk factor for glucose intolerance during pregnancy [12, 40], explaining the increased risk of GDM in women with a prepregnant BMI ≥ 25 in their first pregnancy when compared to women with BMI < 25 [Fig 4]. A normal pregnancy is characterized by a 50%–60% physiological decrease in insulin sensitivity (before conception until late pregnancy) [21, 40], and the pancreatic β cells compensate for this by increasing their insulin secretion [12]. Results from our study suggest that increasing weight between first and second pregnancy, during a relatively short time (more than 60% of the women in our study had an interpregnancy interval of <24 months; Table 1), may stress the glucose metabolism and cause a subclinical decreased insulin sensitivity among normal-weight women as well as overweight women. Normal-weight women generally have a higher insulin sensitivity than overweight and obese women [40]. We suggest that an additive effect of the physiological decrease in insulin sensitivity during pregnancy may overload the capacity and increase the susceptibility to develop GDM, especially in normal-weight women who are used to higher insulin sensitivity.

### Strength and limitations

We used data from a large population-based register with compulsory notification and negligible selection bias. Data were family-based, collected prospectively with a longitudinal design and included several confounding factors that made it possible to evaluate weight change as a causal mechanism behind GDM. BMI represents prepregnant BMI and is not obscured by an effect of gestational weight gain upon the risk of GDM, in contrast to most studies reporting BMI before week 15 of pregnancy [25, 38, 39] or week 16–17 [36]. A limitation in our study was the limited proportion of information on BMI available in both first and second pregnancy during the study period. However, comparing the study population with the population with missing information on BMI [S10 Table], our study population is likely to be representative. Maternal weight gain during pregnancy depends on prepregnant weight [22]. When evaluating effect modification by gestational weight gain, we stratified on a possible intermediate variable on the causal path from prepregnant BMI in second pregnancy (part of our exposure) and GDM, which may have introduced bias in our estimates [41]. We cannot exclude unmeasured confounding, as we were not able to take into account other time-varying variables associated with GDM, such as family history of GDM, mothers weight gain in childhood/early adulthood, prepregnant diet, and vigorous exercise [34]. Data on height and weight are self-reported, and as height tends to be overreported whereas weight is under-reported, this might cause bias due to misclassification [42]. However, several studies have found self-reported height and weight in women of reproductive age and in pregnant women to be valid [43, 44]. As data are collected prospectively, misclassification of BMI is independent of a later GDM diagnosis and therefore likely to be nondifferential, which would tend to bias risk estimates toward the null and underestimate the risk of GDM. We did not include gestational weight gain during first pregnancy in our model and were therefore not able to make a distinction between gestational weight gain in first pregnancy and interpregnancy weight change. According to observational studies, gestational weight gain is strongly associated with short- and long-term postpartum weight retention [22, 45].

### Health implications

Pregnant women who increase their weight by ≥1 BMI unit from their first to second pregnancy should be closely monitored during their second pregnancy to reveal development of GDM, irrespective of prepregnant BMI. Antenatal guidelines for monitoring GDM in pregnancy should add interpregnancy weight change as an independent risk factor for GDM with a routine stress-test of glucose tolerance during pregnancy in women with weight gain more than 1 BMI unit. A possible preventive effect on GDM of losing weight between pregnancies in overweight women needs to be replicated in other studies. Efforts that are targeting women who are overweight in pregnancy and childbirth should expand in focus to promote healthy weight from preconception throughout reproduction. Today, less than 10% of countries’ national policies address healthy maternal weight across the entire spectrum of childbearing [46].

## Conclusion

Women who increased their weight by ≥1 BMI unit from first to second pregnancy had increased risk of GDM in the second pregnancy compared to women with stable weight (−1 to <1 BMI unit change). This applied to women with prepregnant BMI below and above 25 in the first pregnancy; however, the strongest association was found in women with BMI < 25. We found a preventive effect on GDM in overweight women (first pregnancy) who reduced their weight by ≥2 BMI units until the second pregnancy. Our results support a metabolic mechanism behind the increased risk of GDM, represented by the weight change itself.

## Supporting information

S1 STROBE statement (DOC)

S1 Analysis plan (DOCX)

S1 Fig Statistics from the Medical Birth Registry of Norway showing percent (%) of women who have their Body Mass Index (BMI) reported (grey columns) and the proportions of women in the different BMI categories (colored lines) during 2006 to 2014. (TIF)

S1 Table Overall relative risk (RR) for Gestational Diabetes Mellitus (GDM) By interpregnancy change in Body Mass Index (BMI), with and without missing imputation in the adjusted analyses. *All variables from the analysis model, including the outcome variable GDM, were included as imputation variables. In addition, we included the following auxiliary variables in the imputation: prepregnant BMI in second pregnancy, father's education, mother's marital status, birthweight of the child in the second pregnancy, gestational age, preterm birth, preeclampsia or hypertension in pregnancy, and placenta abruptio. The number of imputations was set to 20. **All models are adjusted for maternal age during second pregnancy (<25 [reference], 25–29, 30–34, ≥35 years), maternal country of birth (Nordic [reference]/non-Nordic), maternal education (<11, 11–13, ≥14 [reference] years), interpregnancy interval (<12, 12–23 [reference], 24–35, ≥36 months), and year of second birth (continuous) in addition to smoking during second pregnancy. a) Adjusted analysis without missing imputation. Include only cases with complete information on smoking, education, and maternal country of birth. b) Missing imputation on smoking and education. c) Missing imputation on smoking, education, and maternal country of birth. The imputation allows computed values to be used in the imputation of another variable.
(DOCX)

S2 Table Risk for Gestational Diabetes Mellitus (GDM) by interpregnancy change in Body Mass Index (BMI), stratified by prepregnant BMI in first pregnancy (*n* = 24,198), the Medical Birth Registry of Norway 2006–2014. *Adjusted for maternal age in second pregnancy (<25 [reference], 25–29, 30–34, ≥35 years), maternal country of birth (Nordic [reference]/non-Nordic), maternal education (<11, 11–13, ≥14 [reference] years), smoking in pregnancy (no [reference]/yes), interpregnancy interval (<12, 12–23 [reference], 24–35, ≥36 months), and year of second birth (continuous).
(DOCX)

S3 Table Relative risk (RR) for Gestational Diabetes Mellitus (GDM) by interpregnancy change in Body Mass Index (BMI), stratified by prepregnant BMI in first pregnancy; adjusted analyses with and without missing imputation. *All variables from the analysis model, including the outcome variable GDM, were included as imputation variables. In addition, we included the following auxiliary variables in the imputation: prepregnant BMI in second pregnancy, father's education, mother's marital status, birthweight of the child in the second pregnancy, gestational age, preterm birth, preeclampsia or hypertension in pregnancy, and placenta abruptio. The number of imputations was set to 20. **All models are adjusted for maternal age in second pregnancy (<25 [reference], 25–29, 30–34, ≥35 years), maternal country of birth (Nordic [reference]/non-Nordic), maternal education (<11, 11–13, ≥14 [reference] years), interpregnancy interval (<12, 12–23 [reference], 24–35, ≥36 months), and year of second birth (continuous) in addition to smoking in second pregnancy. a) Adjusted analysis without missing imputation. Include only cases with complete information on smoking, education, and maternal country of birth. b) Missing imputation on smoking and education. c) Missing imputation on smoking, education and maternal country of birth. The imputation allows computed values to be used in the imputation of another variable.
(DOCX)

S4 Table A. Overall relative risk (RR) for Gestational Diabetes Mellitus (GDM) by interpregnancy change in Body Mass Index (BMI) (*n* = 22,443). *When excluding second pregnancies with gestational age below 37 weeks, multiple pregnancies, and pregnancies with hypertensive disorders in second pregnancy (hypertension during pregnancy, preeclampsia, eclampsia, and HELLP). **Adjusted for maternal age in second pregnancy (<25 [reference], 25–29, 30–34, ≥35 years), maternal country of birth (Nordic [reference]/non-Nordic), maternal education (<11, 11–13, ≥14 [reference] years), smoking in pregnancy (no[reference]/yes), interpregnancy interval (<12, 12–23 [reference], 24–35, ≥36 months), and year of second birth (continuous). B. RR for GDM by interpregnancy change in BMI, stratified by prepregnant BMI in first pregnancy. *When excluding second pregnancies with gestational age below 37 weeks, multiple pregnancies and pregnancies with hypertensive disorders in second pregnancy (hypertension during pregnancy, preeclampsia, eclampsia, and HELLP). **Adjusted for maternal age in second pregnancy (<25 [reference], 25–29, 30–34, ≥35 years), maternal country of birth (Nordic [reference]/non-Nordic), maternal education (<11, 11–13, ≥14 [reference] years), smoking in pregnancy (no [reference]/yes), interpregnancy interval (<12, 12–23 [reference], 24–35, ≥36 months), and year of second birth (continuous).
(DOCX)

S5 Table Relative risk (RR) for Gestational Diabetes Mellitus (GDM) in second pregnancy by interpregnancy change in Body Mass Index (BMI), stratified by maternal country of birth (*n* = 24,043), the Medical Birth Registry of Norway. *Adjusted for maternal age in second pregnancy (<25 [reference], 25–29, 30–34, ≥35 years), maternal education (<11, 11–13, ≥14 [reference] years), smoking in pregnancy (no [reference]/yes), interpregnancy interval (<12, 12–23 [reference], 24–35, ≥36 months), and year of second birth (continuous).
(DOCX)

S6 Table Relative risk (RR) for Gestational Diabetes Mellitus (GDM) in second pregnancy by interpregnancy change in Body Mass Index (BMI), stratified by maternal age at second pregnancy (*n* = 24,198), the Medical Birth Registry of Norway. *Adjusted for maternal country of birth (Nordic [reference]/non-Nordic), maternal education (<11, 11–13, ≥14 [reference] years), smoking in pregnancy (no[reference]/yes), interpregnancy interval (<12, 12–23 [reference], 24–35, ≥36 months), and year of second birth (continuous).
(DOCX)

S7 Table Relative risk (RR) for Gestational Diabetes Mellitus (GDM) in second pregnancy by interpregnancy change in Body Mass Index (BMI), stratified by interpregnancy interval (*n* = 24,169), the Medical Birth Registry of Norway. *Adjusted for maternal age in second pregnancy (<25 [reference], 25–29, 30–34, ≥35 years), maternal country of birth (Nordic [reference]/non-Nordic), maternal education (<11, 11–13, ≥14 [reference] years), smoking in pregnancy (no [reference]/yes), 24–35, ≥36 months), and year of second birth (continuous).
(DOCX)

S8 Table Relative risk (RR) for Gestational Diabetes Mellitus (GDM) in second pregnancy by interpregnancy change in Body Mass Index (BMI), in women with BMI < 25 in both pregnancies (*n* = 14,857), the Medical Birth Registry of Norway. *Adjusted for maternal age in second pregnancy (<25 [reference], 25–29, 30–34, ≥35 years), maternal country of birth (Nordic [reference]/non-Nordic), maternal education (<11, 11–13, ≥14 [reference] years), smoking in pregnancy (no[reference]/yes), interpregnancy interval (<12, 12–23 [reference], 24–35, ≥36 months), and year of second birth (continuous). **Adjusted analyses with missing imputation on maternal smoking, education and country of birth.
(DOCX)

S9 Table Relative risk (RR) for Gestational Diabetes Mellitus (GDM) in second pregnancy by interpregnancy change in Body Mass Index (BMI), stratified by prepregnant BMI in second pregnancy (*n* = 24,198), the Medical Birth Registry of Norway 2006–2014. *Adjusted (a) for maternal age in second pregnancy (<25 [reference], 25–29, 30–34, ≥35 years), maternal country of birth (Nordic [reference]/non-Nordic), maternal education (<11, 11–13, ≥14 [reference] years), smoking in pregnancy (no [reference],/yes), interpregnancy interval (<12, 12–23 [reference], 24–35, ≥36 months), and year of second birth (continuous).
(DOCX)

S10 Table The study population (*n* = 24,198) compared to the population with missing information on prepregnant Body Mass Index (BMI) in first and second pregnancy (*n* = 79,284). *Mothers with first and second pregnancy between 2006–2014, without Gestational Diabetes Mellitus (GDM) in first pregnancy and without diabetes mellitus prior to first and second pregnancy.
(DOCX)

## Abbreviations

a: adjusted
BMI: Body Mass Index
GDM: Gestational Diabetes Mellitus
MBRN: Medical Birth Registry of Norway
RR: relative risk
HELLP: Hemolysis Elevated Liver enzymes and Low Platelet count
