# Supplementary material for: Gestational diabetes mellitus and interpregnancy weight change: A population-based cohort study
Source: PLoS Med. 2017 Aug 1;14(8):e1002367. doi: 10.1371/journal.pmed.1002367 (PMC5538633; doi:10.1371/journal.pmed.1002367)
Supplement: S1 STROBE statement — (DOC) [file pmed.1002367.s001.doc]

**STROBE Statement**—Checklist of items that should be included in reports of ***cohort studies***

Corresponding Author: Linn Marie Sorbye

|  | Item No | Recommendation | Author’s Response |
| --- | --- | --- | --- |
| **Title and abstract** | 1 | (*a*) Indicate the study’s design with a commonly  used term in the title or the abstract | See Title and “Methods and Findings” in the Abstract. |
|  |  | (*b*) Provide in the abstract an informative and  balanced summary of what was done and what  was found | See “Background” and “Methods and Findings” in the Abstract. |
| Introduction | | |  |
| Background/rationale | 2 | Explain the scientific background and rationale  for the investigation being reported | See Introduction, paragraph 2. |
| Objectives | 3 | State specific objectives, including any  prespecified hypotheses | See Introduction, paragraph 2. |
| Methods | | |  |
| Study design | 4 | Present key elements of study design early in  the paper | See Title, “Methods and Findings” in the Abstract and “Data sources” in Methods, paragraph 1. |
| Setting | 5 | Describe the setting, locations, and relevant dates,  including periods of recruitment, exposure, follow-up,  and data collection | See “Data sources” in Methods, paragraph 1. |
| Participants | 6 | (*a*) Give the eligibility criteria, and the sources and methods of  selection of participants. Describe methods of follow-up | See “Inclusions and definitions” in Methods, paragraph 1 and Fig1. |
|  |  | (*b*) For matched studies, give matching criteria and number of  exposed and unexposed |  |
| Variables | 7 | Clearly define all outcomes, exposures, predictors, potential  confounders, and effect modifiers. Give diagnostic criteria,  if applicable | See “Inclusions and definitions”, in Methods, paragraph 1 and 2. Diagnostic criteria see “Inclusions and definitions”, paragraph 2. |
| Data sources/ measurement | 8* | For each variable of interest, give sources of data and details  of methods of assessment (measurement). Describe  comparability of assessment methods if there is more than one group | See “Data sources” and “Inclusions and definitions”, paragraph 1 and 2. |
| Bias | 9 | Describe any efforts to address potential sources of bias |  |
| Study size | 10 | Explain how the study size was arrived at | See “Inclusions and definitions”, paragraph 1 and Fig1. |
| Quantitative variables | 11 | Explain how quantitative variables were handled in the analyses.  If applicable, describe which groupings were chosen and why | See “Inclusions and definitions”, paragraph 1 and 2. See Table1 |
| Statistical methods | 12 | (*a*) Describe all statistical methods, including those used to control  for confounding | See “Statistics”. Missing imputation described in “Statistics”. See stratified analyses S5Table, S6Table and S7Table. |
|  |  | (*b*) Describe any methods used to examine subgroups and interactions | See “Pre-pregnant BMI in first Pregnancy, inter-pregnancy weight change and GDM” in Findings, paragraph 2. |
|  |  | (*c*) Explain how missing data were addressed | See “Statistics” |
|  |  | (*d*) If applicable, explain how loss to follow-up was addressed |  |
|  |  | (*e*) Describe any sensitivity analyses | See “Pre-pregnant BMI in first Pregnancy, inter-pregnancy weight change and GDM” in Findings, paragraph 2. |
| Results | | |  |
| Participants | 13* | (a) Report numbers of individuals at each stage of study—eg numbers potentially eligible, examined for eligibility, confirmed eligible, included in the study, completing follow-up, and analysed | See “Inclusions and definitions” in Methods, paragraph 1 and  Fig1 shows the flow-chart. |
|  |  | (b) Give reasons for non-participation at each stage | See “Inclusions and definitions” in Methods, paragraph 1. |
|  |  | (c) Consider use of a flow diagram | See Fig1. |
| Descriptive data | 14* | (a) Give characteristics of study participants (eg demographic, clinical, social) and information on exposures and potential confounders | See Table1. |
|  |  | (b) Indicate number of participants with missing data for each variable of interest | See Table1. |
|  |  | (c) Summarise follow-up time (eg, average and total amount) |  |
| Outcome data | 15* | Report numbers of outcome events or summary measures over time | See Table1. |
| Main results | 16 | (*a*) Give unadjusted estimates and, if applicable, confounder-adjusted estimates and their precision (eg, 95% confidence interval). Make clear which confounders were adjusted for and why they were included | See all Figures, Tables and Supplementary Tables |
|  |  | (*b*) Report category boundaries when continuous variables were categorized |  |
|  |  | (*c*) If relevant, consider translating estimates of relative risk into absolute risk for a meaningful time period | See additional information on absolute risk in Tables. |
| Other analyses | 17 | Report other analyses done—eg analyses of subgroups and interactions, and sensitivity analyses | See all Supplementary files |
| Discussion | | |  |
| Key results | 18 | Summarise key results with reference to study objectives | See “Principal findings” and “Inter-pregnancy weight change, BMI in first pregnancy and the risk of GDM” in the Discussion. |
| Limitations | 19 | Discuss limitations of the study, taking into account sources of potential bias or imprecision. Discuss both direction and magnitude of any potential bias | See “Strength and limitations” in the Discussion. |
| Interpretation | 20 | Give a cautious overall interpretation of results considering objectives, limitations, multiplicity of analyses, results from similar studies, and other relevant evidence | See the six different paragraphs of the Discussion. |
| Generalisability | 21 | Discuss the generalisability (external validity) of the study results | See “Health Implications”. |
| Other information | | |  |
| Funding | 22 | Give the source of funding and the role of the funders for the present study and, if applicable, for the original study on which the present article is based | See information in the “Financial Disclosure” field in the submission form. |

*Give information separately for exposed and unexposed groups.

**Note:** An Explanation and Elaboration article discusses each checklist item and gives methodological background and published examples of transparent reporting. The STROBE checklist is best used in conjunction with this article (freely available on the Web sites of PLoS Medicine at http://www.plosmedicine.org/, Annals of Internal Medicine at http://www.annals.org/, and Epidemiology at http://www.epidem.com/). Information on the STROBE Initiative is available at http://www.strobe-statement.org.
