# Supplementary material for: Gestational diabetes mellitus and interpregnancy weight change: A population-based cohort study
Source: PLoS Med. 2017 Aug 1;14(8):e1002367. doi: 10.1371/journal.pmed.1002367 (PMC5538633; doi:10.1371/journal.pmed.1002367)

**Analysis plan** June 2016

___________________________________________________________________________

**Study title:** Inter-pregnancy Weight Change, Gestational Weight Gain and Gestational Diabetes Mellitus

**Investigators:** Linn Marie Sørbye, Rolv Skjærven (Project leader), Kari Klungsøyr and Nils-Halvdan Morken (Main supervisor)

**Research group:** Reproductive Epidemiology with a Life-Course Perspective, Department of Global Public Health and Primary Care, University of Bergen, 5020 Bergen, Norway.

**Ethics:** The project is approved by the Regional Ethics Committee, Western region of Norway (REK VEST 2015/1728). Data are de-identified in accordance with the Medical Birth Registry of Norway (MBRN) Regulations and the Privacy Act. We aim to follow the guidelines of the Declaration of Helsinki.

**Hypothesis:**

There is an association between inter-pregnancy weight change from first to second pregnancy and the risk of Gestational Diabetes Mellitus in second pregnancy. The association depends on pre-pregnant Body Mass Index (BMI) in first pregnancy and gestational weight gain in second pregnancy.

**Specific Aims:**

1. Investigate the risk of Gestational Diabetes Mellitus in Second Pregnancy by inter-pregnancy weight change from first to second pregnancy
2. Investigate pre-pregnant BMI in first pregnancy and gestational weight gain in second pregnancy as effect modification variables

**Study design:** Observational Cohort Study using family-design.

**Data Sources:** Data from the population-based Norwegian Medical Birth Registry. Data are prospectively recorded. Each birth is linked to its mother by the unique identification number.

**Study Population:** Mothers with their first and second pregnancy during 2006-2014.

**Varables:**

Main exposure: Inter-pregnancy weight change from first to second pregnancy (BMI units)

Main outcome: Gestational Diabetes Mellitus in second pregnancy (yes/no)

Effect modifying variables: Pre-pregnant BMI in first pregnancy and gestational weight gain (weight loss, 0-7.9, 8-15.9 kilo and ≥16 kilo) in second pregnancy

Possible confounders: Maternal age in second pregnancy, maternal country of birth, maternal education, maternal smoking in second pregnancy, inter-pregnancy interval and year of second birth.

**Statistics:** Chi-square test will be used to investigate associations and linear trends between categorical variables. To estimate the association between categories of inter-pregnancy weight change and the risk for Gestational Diabetes Mellitus we will use general linear models with extension for the binary family in STATA. Relative risk with 95% confidence interval will be made for each of the inter-pregnancy weight change categories.

Statistical analyses will be performed using STATA IC Statistical software version 14 and Statistical Package for the Social Sciences version 23 (SPSS).

**During the process** April 2017

The analysis plan was made prior to the analyses.

Stratified analyses: In addition to adjusting for confounders in the multiplicative model, we also stratified the analyses according to inter-pregnancy interval (<24 and ≥24 months), mothers country of birth (Nordic, non-Nordic women), smoking in second pregnancy (yes, no), education (<11, 11-13, ≥14 years), maternal age at the second pregnancy (<30, ≥30 years) and mothers height (<1.68, ≥1.68 cm); The association between inter-pregnancy weight change (<-2, -2 to <-1, -1 to <1 (reference), 1 to <2, 2 to <4 and ≥4 BMI units) and risk of Gestational Diabetes Mellitus revealed the same pattern in the stratified analyses as in the overall analysis.

Sensitivity analyses: We performed sensitivity analyses where we excluded pregnancies with gestational age below 28 or 36 weeks, multiple pregnancies or pregnancies with hypertensive disorders in second pregnancy (hypertension during pregnancy, preeclampsia, HELLP), and results remained unchanged.

Comparing populations: Due to missing cases of inter-pregnancy weight change, we compared the study population with the population that had missing data.

Interaction analyses: To evaluate effect modification by pre-pregnant BMI in first pregnancy (<25, ≥25) and gestational weight gain in second pregnancy (<14, ≥14 kg), we included the interaction term in the multiplicative model evaluated by Likelihood ratio test in STATA. Due to limited cases we were not able to look at gestational weight gain in more than 2 categories when evaluating the effect modifying role of gestational weight gain in second pregnancy. We decided to use the median value as threshold when categorising gestational weight gain (<14, ≥14 kg).


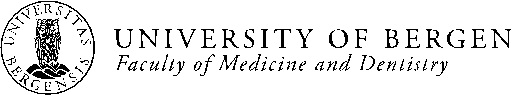


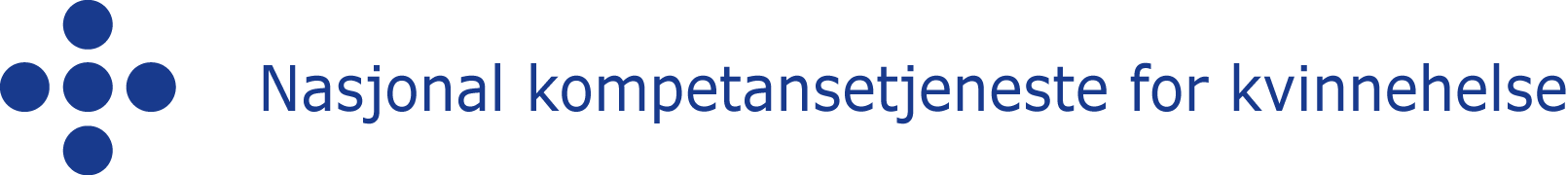


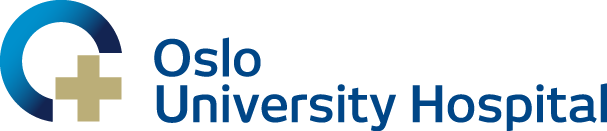

Supplement: S1 Analysis plan — (DOCX) [file pmed.1002367.s002.docx]
