# Supplementary material for: Gestational diabetes mellitus and interpregnancy weight change: A population-based cohort study
Source: PLoS Med. 2017 Aug 1;14(8):e1002367. doi: 10.1371/journal.pmed.1002367 (PMC5538633; doi:10.1371/journal.pmed.1002367)
Supplement: S1 Table — *All variables from the analysis model, including the outcome variable GDM, were included as imputation variables. In addition, we included the following auxiliary variables in the imputation: prepregnant BMI in second pregnancy, father's education, mother's marital status, birthweight of the child in the second pregnancy, gestational age, preterm birth, preeclampsia or hypertension in pregnancy, and placenta abruptio. The number of imputations was set to 20. **All models are adjusted for maternal age during second pregnancy (<25 [reference], 25–29, 30–34, ≥35 years), maternal country of birth (Nordic [reference]/non-Nordic), maternal education (<11, 11–13, ≥14 [reference] years), interpregnancy interval (<12, 12–23 [reference], 24–35, ≥36 months), and year of second birth (continuous) in addition to smoking during second pregnancy. a) Adjusted analysis without missing imputation. Include only cases with complete information on smoking, education, and maternal country of birth. b) Missing imputation on smoking and education. c) Missing imputation on smoking, education, and maternal country of birth. The imputation allows computed values to be used in the imputation of another variable. (DOCX) [file pmed.1002367.s004.docx]

**S1 Table. Overall relative risk (RR) for Gestational Diabetes Mellitus (GDM) By interpregnancy change in Body Mass Index (BMI), with and without missing imputation in the adjusted (a) analyses.**

| **BMI Change Units (kg/m^2^)** |  |  | **Relative risk for GDM in second pregnancy** | | | | | | |
| --- | --- | --- | --- | --- | --- | --- | --- | --- | --- |
|  | **N** | **GDM/1000** |  |  | **Model a)** |  | **Model b)*** |  | **Model c)*** |
|  |  |  | **Crude RR** |  | **a RR**** |  | **a RR**** |  | **a RR**** |
| **< -2** | 15/1,692 | 8.9 | 0.81 |  | 0.89 |  | 0.86 |  | 0.85 |
| **-2 til < -1** | 34/2,384 | 14.3 | 1.30 |  | 1.31 |  | 1.32 |  | 1.35 |
| **-1 til < 1** | 126/11,512 | 10.9 | 1.00 |  | 1.00 |  | 1.00 |  | 1.00 |
| **1 til < 2** | 79/3,814 | 20.7 | 1.89 |  | 1.96 |  | 1.83 |  | 1.82 |
| **2 til < 4** | 97/3,279 | 29.6 | 2.70 |  | 2.64 |  | 2.54 |  | 2.55 |
| **≥ 4** | 88/1,517 | 58.0 | 5.30 |  | 5.44 |  | 5.06 |  | 5.05 |
| **Total** | 439/24,198 | 18.1 | 24,198 |  | 20,824 |  | 23,686 |  | 23,837 |

*All variables from the analysis model, including the outcome variable GDM, were included as imputation variables. In addition, we included the following auxiliary variables in the imputation: prepregnant BMI in second pregnancy, father's education, mother's marital status, birthweight of the child in the second pregnancy, gestational age, preterm birth, preeclampsia or hypertension in pregnancy, and placenta abruptio. The number of imputations was set to 20.

**All models are adjusted for maternal age during second pregnancy (<25 [reference], 25–29, 30–34, ≥35 years), maternal country of birth (Nordic [reference]/non-Nordic), maternal education (<11, 11–13, ≥14 [reference] years), interpregnancy interval (<12, 12–23 [reference], 24–35, ≥36 months), and year of second birth (continuous) in addition to smoking during second pregnancy.

a) Adjusted analysis without missing imputation. Include only cases with complete information on smoking, education, and maternal country of birth.

b) Missing imputation on smoking and education.

c) Missing imputation on smoking, education, and maternal country of birth. The imputation allows computed values to be used in the imputation of another variable.

.
