# Supplementary material for: Gestational diabetes mellitus and interpregnancy weight change: A population-based cohort study
Source: PLoS Med. 2017 Aug 1;14(8):e1002367. doi: 10.1371/journal.pmed.1002367 (PMC5538633; doi:10.1371/journal.pmed.1002367)
Supplement: S2 Table — *Adjusted for maternal age in second pregnancy (<25 [reference], 25–29, 30–34, ≥35 years), maternal country of birth (Nordic [reference]/non-Nordic), maternal education (<11, 11–13, ≥14 [reference] years), smoking in pregnancy (no [reference]/yes), interpregnancy interval (<12, 12–23 [reference], 24–35, ≥36 months), and year of second birth (continuous). (DOCX) [file pmed.1002367.s005.docx]

**S2 Table. Risk for Gestational Diabetes Mellitus (GDM) by interpregnancy change in Body Mass Index (BMI), stratified by prepregnant BMI in first pregnancy (*n* = 24,198), the Medical Birth Registry of Norway 2006–2014.**

| **BMI Change**  **Units kg/m2** | **Prepregnant BMI < 25 first pregnancy** | | | | | |  |  | **Prepregnant BMI ≥ 25 in first pregnancy** | | | | |
| --- | --- | --- | --- | --- | --- | --- | --- | --- | --- | --- | --- | --- | --- |
|  | **N** | **GDM /1000** | **Crude RR** | **95% CI** | **a RR*** | **95% CI** |  | **N** | **GDM**  **/1000** | **Crude**  **RR** | **95% CI** | **a RR*** | **95% CI** |
| **< -2** | 4/600 | 6.7 | 1.1 | 0.4-2.9 | 1.0 | 0.3-3.3 |  | 11/1,092 | 10.1 | 0.3 | 0.2-0.7 | 0.4 | 0.2-0.8 |
| **-2 to <-1** | 13/1,611 | 8.1 | 1.3 | 0.7-2.4 | 1.1 | 0.6-2.3 |  | 21/773 | 27.2 | 0.9 | 0.6-1.5 | 0.9 | 0.6-1.7 |
| **-1 to < 1** | 57/9,156 | 6.2 | 1.0 | Reference | 1.0 | Reference |  | 69/2,356 | 29.3 | 1.0 | Reference | 1.0 | Reference |
| **1 to < 2** | 36/2,788 | 12.9 | 2.1 | 1.4-3.1 | 2.0 | 1.2-3.1 |  | 43/1,026 | 41.9 | 1.4 | 0.98-2.1 | 1.6 | 1.1-2.4 |
| **2 to < 4** | 33/2,143 | 15.4 | 2.5 | 1.6-3.8 | 2.1 | 1.3-3.4 |  | 64/1,136 | 56.3 | 1.9 | 1.4-2.7 | 2.0 | 1.4-2.9 |
| **≥4** | 39/819 | 47.6 | 7.7 | 5.1-11.4 | 7.2 | 4.5-11.3 |  | 49/698 | 70.2 | 2.4 | 1.7-3.4 | 2.6 | 1.7-3.8 |
| **Total** | 182/17,117 | 10.6 |  |  | 14,677 |  |  | 257/7,081 | 36.3 |  |  | 6,147 |  |

*Adjusted (a) for maternal age in second pregnancy (<25 [reference], 25–29, 30–34, ≥35 years), maternal country of birth (Nordic [reference]/non-Nordic), maternal education (<11, 11–13, ≥14 [reference] years), smoking in pregnancy (no [reference]/yes), interpregnancy interval (<12, 12–23 [reference], 24–35, ≥36 months), and year of second birth (continuous).
