# Supplementary material for: Gestational diabetes mellitus and interpregnancy weight change: A population-based cohort study
Source: PLoS Med. 2017 Aug 1;14(8):e1002367. doi: 10.1371/journal.pmed.1002367 (PMC5538633; doi:10.1371/journal.pmed.1002367)
Supplement: S4 Table — A. Overall relative risk (RR) for Gestational Diabetes Mellitus (GDM) by interpregnancy change in Body Mass Index (BMI) (n = 22,443). *When excluding second pregnancies with gestational age below 37 weeks, multiple pregnancies, and pregnancies with hypertensive disorders in second pregnancy (hypertension during pregnancy, preeclampsia, eclampsia, and HELLP). **Adjusted for maternal age in second pregnancy (<25 [reference], 25–29, 30–34, ≥35 years), maternal country of birth (Nordic [reference]/non-Nordic), maternal education (<11, 11–13, ≥14 [reference] years), smoking in pregnancy (no[reference]/yes), interpregnancy interval (<12, 12–23 [reference], 24–35, ≥36 months), and year of second birth (continuous). B. RR for GDM by interpregnancy change in BMI, stratified by prepregnant BMI in first pregnancy. *When excluding second pregnancies with gestational age below 37 weeks, multiple pregnancies and pregnancies with hypertensive disorders in second pregnancy (hypertension during pregnancy, preeclampsia, eclampsia, and HELLP). **Adjusted for maternal age in second pregnancy (<25 [reference], 25–29, 30–34, ≥35 years), maternal country of birth (Nordic [reference]/non-Nordic), maternal education (<11, 11–13, ≥14 [reference] years), smoking in pregnancy (no [reference]/yes), interpregnancy interval (<12, 12–23 [reference], 24–35, ≥36 months), and year of second birth (continuous). (DOCX) [file pmed.1002367.s007.docx]

**S4 Table. A. Overall relative risk (RR) for Gestational Diabetes Mellitus (GDM) by interpregnancy change in Body Mass Index (BMI) (*n* = 22,443).**

| **Interpregnancy BMI change** | **Risk for Gestational Diabetes Mellitus*** | | | | | | |
| --- | --- | --- | --- | --- | --- | --- | --- |
|  | **Cases GDM** | **Total** | **Crude RR** | **95% CI** |  | **a RR**** | **95% CI** |
| **<-2** | 14 | 1,575 | 0.87 | 0.50-1.51 |  | 0.96 | 0.53-1.75 |
| **-2 til < - 1** | 33 | 2,201 | 1.46 | 0.99-2.16 |  | 1.52 | 0.99-2.32 |
| **-1 til < 1** | 110 | 10,745 | 1.00 | Reference |  | 1.00 | Reference |
| **1 til <2** | 72 | 3,540 | 1.99 | 1.48-2.67 |  | 2.13 | 1.56-2.93 |
| **2 til <4** | 83 | 3,010 | 2.69 | 2.03-3.57 |  | 2.59 | 1.89-3.55 |
| **3.5≥4** | 81 | 1,372 | 5.77 | 4.35-7.64 |  | 5.87 | 4.27-8.06 |
| **Total** | 393 | 22,443 | 22,443 | |  | 19,300 | |

*When excluding second pregnancies with gestational age below 37 weeks, multiple pregnancies, and pregnancies with hypertensive disorders in second pregnancy (hypertension during pregnancy, preeclampsia, eclampsia, and HELLP(Hemolysis Elevated Liver enzymes and Low Platelet count)).

**Adjusted (a) for maternal age in second pregnancy (<25 [reference], 25–29, 30–34, ≥35 years), maternal country of birth (Nordic [reference]/non-Nordic), maternal education (<11, 11–13, ≥14 [reference] years), smoking in pregnancy (no[reference]/yes), interpregnancy interval (<12, 12–23 [reference], 24–35, ≥36 months), and year of second birth (continuous).

**S4 Table. B Relative risk (RR) for Gestational Diabetes Mellitus (GDM) by interpregnancy change in Body Mass Index (BMI) (*n* = 22,443), stratified by prepregnant BMI in first pregnancy.**

| **BMI Change**  **Units kg/m2** | **Pre-pregnant BMI <25 in first pregnancy*** | | | | | **Pre-pregnant BMI ≥25 in first pregnancy*** | | | | |
| --- | --- | --- | --- | --- | --- | --- | --- | --- | --- | --- |
|  | **Crude RR** | **95% CI** | **a RR**** | **95% CI** |  | | **Crude**  **RR** | **95% CI** | **a RR**** | **95% CI** |
| **<-2** | 1.19 | 0.43-3.28 | 1.19 | 0.37-3.85 |  | | 0.36 | 0.19-0.70 | 0.41 | 0.20-0.83 |
| **-2 to < - 1** | 1.47 | 0.80-2.69 | 1.33 | 0.65-2.72 |  | | 1.03 | 0.63-1.70 | 1.13 | 0.66-1.92 |
| **-1 to < 1** | 1.00 | Reference | 1.00 | Reference |  | | 1.00 | Reference | 1.00 | Reference |
| **1 to <2** | 2.20 | 1.43-3.39 | 2.16 | 1.35-3.46 |  | | 1.48 | 0.99-2.20 | 1.72 | 1.13-2.61 |
| **2 to <4** | 2.37 | 1.50-3.74 | 2.02 | 1.19-3.43 |  | | 1.97 | 1.37-2.82 | 1.96 | 1.32-2.90 |
| **≥4** | 7.79 | 5.10-11.90 | 7.29 | 4.49-11.83 |  | | 2.72 | 1.87-3.96 | 2.83 | 1.87-4.28 |
| **Total** | 16,022 |  | 13,723 |  |  | | 6,421 |  | 5,577 |  |

*When excluding second pregnancies with gestational age below 37 weeks, multiple pregnancies and pregnancies with hypertensive disorders in second pregnancy (hypertension during pregnancy, preeclampsia, eclampsia, and HELLP).

**Adjusted (a) for maternal age in second pregnancy (<25 [reference], 25–29, 30–34, ≥35 years), maternal country of birth (Nordic [reference]/non-Nordic), maternal education (<11, 11–13, ≥14 [reference] years), smoking in pregnancy (no [reference]/yes), interpregnancy interval (<12, 12–23 [reference], 24–35, ≥36 months), and year of second birth (continuous).
