# Supplementary material for: Gestational diabetes mellitus and interpregnancy weight change: A population-based cohort study
Source: PLoS Med. 2017 Aug 1;14(8):e1002367. doi: 10.1371/journal.pmed.1002367 (PMC5538633; doi:10.1371/journal.pmed.1002367)
Supplement: S5 Table — *Adjusted for maternal age in second pregnancy (<25 [reference], 25–29, 30–34, ≥35 years), maternal education (<11, 11–13, ≥14 [reference] years), smoking in pregnancy (no [reference]/yes), interpregnancy interval (<12, 12–23 [reference], 24–35, ≥36 months), and year of second birth (continuous). (DOCX) [file pmed.1002367.s008.docx]

**S5 Table. Relative risk (RR) for Gestational Diabetes Mellitus (GDM) in second pregnancy by interpregnancy change in Body Mass Index (BMI), stratified by maternal country of birth (*n* = 24,043), the Medical Birth Registry of Norway.**

| **BMI Change**  **Units kg/m2** | **Nordic women** | | | | | **Non-Nordic women** | | | | |
| --- | --- | --- | --- | --- | --- | --- | --- | --- | --- | --- |
|  | **Crude RR** | **95% CI** | **a RR*** | **95% CI** |  | | **Crude**  **RR** | **95% CI** | **a RR*** | **95% CI** |
| **<-2** | 0.82 | 0.45-1.50 | 0.79 | 0.41-1.52 |  | | 0.80 | 0.25-2.63 | 1.36 | 0.40-4.60 |
| **-2 to < - 1** | 1.27 | 0.83-1.96 | 1.24 | 0.78-1.97 |  | | 1.29 | 0.57-2.94 | 1.54 | 0.58-4.11 |
| **-1 to < 1** | 1.00 | Reference | 1.00 | Reference |  | | 1.00 | Reference | 1.00 | Reference |
| **1 to <2** | 1.99 | 1.45-2.73 | 1.97 | 1.41-2.75 |  | | 1.55 | 0.85-2.82 | 1.94 | 0.95-3.96 |
| **2 to <4** | 2.55 | 1.86-3.48 | 2.46 | 1.76-3.45 |  | | 2.66 | 1.61-4.41 | 3.30 | 1.78-6.11 |
| **≥4** | 4.74 | 3.41-6.58 | 4.61 | 3.22-6.60 |  | | 5.55 | 3.40-9.06 | 7.74 | 4.27-14.02 |
| **Total** | 19,828 |  | 17,707 |  |  | | 4,215 |  | 3,117 |  |

*Adjusted (a) for maternal age in second pregnancy (<25 [reference], 25–29, 30–34, ≥35 years), maternal education (<11, 11–13, ≥14 [reference] years), smoking in pregnancy (no [reference]/yes), interpregnancy interval (<12, 12–23 [reference], 24–35, ≥36 months), and year of second birth (continuous).
