# Supplementary material for: Gestational diabetes mellitus and interpregnancy weight change: A population-based cohort study
Source: PLoS Med. 2017 Aug 1;14(8):e1002367. doi: 10.1371/journal.pmed.1002367 (PMC5538633; doi:10.1371/journal.pmed.1002367)
Supplement: S6 Table — *Adjusted for maternal country of birth (Nordic [reference]/non-Nordic), maternal education (<11, 11–13, ≥14 [reference] years), smoking in pregnancy (no[reference]/yes), interpregnancy interval (<12, 12–23 [reference], 24–35, ≥36 months), and year of second birth (continuous). (DOCX) [file pmed.1002367.s009.docx]

**S6 Table. Relative risk (RR) for Gestational Diabetes Mellitus (GDM) in second pregnancy by interpregnancy change in Body Mass Index (BMI), stratified by maternal age at second pregnancy (*n* = 24,198), the Medical Birth Registry of Norway.**

| **BMI Change**  **Units kg/m2** | **Maternal age <30 years** | | | | | **Maternal age ≥30 years** | | | | |
| --- | --- | --- | --- | --- | --- | --- | --- | --- | --- | --- |
|  | **Crude RR** | **95% CI** | **a RR*** | **95% CI** |  | | **Crude**  **RR** | **95% CI** | **a RR*** | **95% CI** |
| **<-2** | 0.57 | 0.20-1.60 | 0.76 | 0.27-2.16 |  | | 1.07 | 0.58-2.0 | 1.01 | 0.51-2.01 |
| **-2 to < - 1** | 1.36 | 0.71-2.61 | 1.36 | 0.64-2.87 |  | | 1.34 | 0.85-2.13 | 1.30 | 0.78-2.15 |
| **-1 to < 1** | 1.00 | Reference | 1.00 | Reference |  | | 1.00 | Reference | 1.00 | Reference |
| **1 to <2** | 2.29 | 1.42-3.69 | 2.77 | 1.64-4.69 |  | | 1.75 | 1.24-2.48 | 1.69 | 1.16-2.46 |
| **2 to <4** | 3.46 | 2.25-5.32 | 3.46 | 2.11-5.67 |  | | 2.49 | 1.78-3.50 | 2.32 | 1.60-3.36 |
| **≥4** | 6.68 | 4.36-10.24 | 7.01 | 4.29-11.44 |  | | 5.20 | 3.64-7.43 | 4.72 | 3.16-7.05 |
| **Total** | 11,490 |  | 9,812 |  |  | | 12,708 |  | 11,012 |  |

*Adjusted (a) for maternal country of birth (Nordic [reference]/non-Nordic), maternal education (<11, 11–13, ≥14 [reference] years), smoking in pregnancy (no[reference]/yes), interpregnancy interval (<12, 12–23 [reference], 24–35, ≥36 months), and year of second birth (continuous).
