# Supplementary material for: Gestational diabetes mellitus and interpregnancy weight change: A population-based cohort study
Source: PLoS Med. 2017 Aug 1;14(8):e1002367. doi: 10.1371/journal.pmed.1002367 (PMC5538633; doi:10.1371/journal.pmed.1002367)
Supplement: S7 Table — *Adjusted for maternal age in second pregnancy (<25 [reference], 25–29, 30–34, ≥35 years), maternal country of birth (Nordic [reference]/non-Nordic), maternal education (<11, 11–13, ≥14 [reference] years), smoking in pregnancy (no [reference]/yes), 24–35, ≥36 months), and year of second birth (continuous). (DOCX) [file pmed.1002367.s010.docx]

**S7 Table. Relative risk (RR) for Gestational Diabetes Mellitus (GDM) in second pregnancy by interpregnancy change in Body Mass Index (BMI), stratified by interpregnancy interval (*n* = 24,169), the Medical Birth Registry of Norway.**

| **BMI Change**  **Units kg/m2** | **Interpregnancy interval < 24 months** | | | | | **Interpregnancy interval ≥ 24 months** | | | | |
| --- | --- | --- | --- | --- | --- | --- | --- | --- | --- | --- |
|  | **Crude RR** | **95% CI** | **a RR*** | **95% CI** |  | | **Crude**  **RR** | **95% CI** | **a RR*** | **95% CI** |
| **<-2** | 1.14 | 0.57-2.29 | 1.14 | 0.52-2.51 |  | | 0.53 | 0.23-1.22 | 0.69 | 0.30-1.61 |
| **-2 to < - 1** | 1.21 | 0.69-2.13 | 1.13 | 0.59-2.17 |  | | 1.43 | 0.86-2.36 | 1.51 | 0.87-2.61 |
| **-1 to < 1** | 1.00 | Reference | 1.00 | Reference |  | | 1.00 | Reference | 1.00 | Reference |
| **1 to <2** | 2.38 | 1.61-3.53 | 2.40 | 1.56-3.70 |  | | 1.43 | 0.96-2.12 | 1.60 | 1.05-2.43 |
| **2 to <4** | 4.25 | 2.99-6.04 | 4.44 | 3.01-6.56 |  | | 1.47 | 0.97-2.21 | 1.40 | 0.88-2.14 |
| **≥4** | 4.99 | 3.27-7.61 | 5.71 | 3.54-9.19 |  | | 4.94 | 3.49-6.98 | 5.07 | 3.46-7.43 |
| **Total** | 15,148 |  | 12,979 |  |  | | 9,021 |  | 7,845 |  |

*Adjusted (a) for maternal age in second pregnancy (<25 [reference], 25–29, 30–34, ≥35 years), maternal country of birth (Nordic [reference]/non-Nordic), maternal education (<11, 11–13, ≥14 [reference] years), smoking in pregnancy (no [reference]/yes), 24–35, ≥36 months), and year of second birth (continuous).
