# Supplementary material for: Gestational diabetes mellitus and interpregnancy weight change: A population-based cohort study
Source: PLoS Med. 2017 Aug 1;14(8):e1002367. doi: 10.1371/journal.pmed.1002367 (PMC5538633; doi:10.1371/journal.pmed.1002367)
Supplement: S8 Table — *Adjusted for maternal age in second pregnancy (<25 [reference], 25–29, 30–34, ≥35 years), maternal country of birth (Nordic [reference]/non-Nordic), maternal education (<11, 11–13, ≥14 [reference] years), smoking in pregnancy (no[reference]/yes), interpregnancy interval (<12, 12–23 [reference], 24–35, ≥36 months), and year of second birth (continuous). **Adjusted analyses with missing imputation on maternal smoking, education and country of birth. (DOCX) [file pmed.1002367.s011.docx]

**S8 Table. Relative risk (RR) for Gestational Diabetes Mellitus (GDM) in second pregnancy by interpregnancy change in Body Mass Index (BMI), in women with BMI < 25 in both pregnancies (*n* = 14,857), the Medical Birth Registry of Norway.**

| **Interpregnancy BMI change (kg/m^2^)** |  | |  | **RR for GDM in second pregnancy** | | | | | |
| --- | --- | --- | --- | --- | --- | --- | --- | --- | --- |
|  | **Total** | **GDM /1000** |  | **Crude RR** | **95% CI** | **a RR*** | **95% CI** | **a RR**** | **95% CI** |
| **<-2** | 4/600 | 6.7 |  | 1.07 | 0.39-2.94 | 1.03 | 0.32-3.32 | 1.19 | 0.43-3.29 |
| **-2 til < - 1** | 13/1,611 | 8.1 |  | 1.30 | 0.71-2.36 | 1.13 | 0.55-2.29 | 1.32 | 0.71-2.46 |
| **-1 til < 1** | 56/8,990 | 6.2 |  | 1.00 | Reference | 1.00 | Reference | 1.00 | Reference |
| **1 til <2** | 26/2,290 | 11.4 |  | 1.82 | 1.15-2.90 | 1.87 | 1.14-3.08 | 1.78 | 1.12-2.85 |
| **2 til <4** | 15/1,216 | 12.3 |  | 1.98 | 1.12-3.49 | 1.79 | 0.94-3.41 | 1.83 | 1.02-3.27 |
| **≥4** | 4/150 | 26.7 |  | 4.28 | 1.57-11.65 | 5.95 | 2.12-16.65 | 4.27 | 1.53-11.94 |
| **Total** | 118/14,857 | 7.9 |  |  |  | 12,771 |  | 14,633 |  |

*Adjusted (a) for maternal age in second pregnancy (<25 [reference], 25–29, 30–34, ≥35 years), maternal country of birth (Nordic [reference]/non-Nordic), maternal education (<11, 11–13, ≥14 [reference] years), smoking in pregnancy (no[reference]/yes), interpregnancy interval (<12, 12–23 [reference], 24–35, ≥36 months), and year of second birth (continuous).

**Adjusted analyses with missing imputation on maternal smoking, education and country of birth.
