# Supplementary material for: Gestational diabetes mellitus and interpregnancy weight change: A population-based cohort study
Source: PLoS Med. 2017 Aug 1;14(8):e1002367. doi: 10.1371/journal.pmed.1002367 (PMC5538633; doi:10.1371/journal.pmed.1002367)
Supplement: S9 Table — *Adjusted (a) for maternal age in second pregnancy (<25 [reference], 25–29, 30–34, ≥35 years), maternal country of birth (Nordic [reference]/non-Nordic), maternal education (<11, 11–13, ≥14 [reference] years), smoking in pregnancy (no [reference],/yes), interpregnancy interval (<12, 12–23 [reference], 24–35, ≥36 months), and year of second birth (continuous). (DOCX) [file pmed.1002367.s012.docx]

**S9 Table. Relative risk (RR) for Gestational Diabetes Mellitus (GDM) in second pregnancy by interpregnancy change in Body Mass Index (BMI), stratified by prepregnant BMI in second pregnancy (*n* = 24,198), the Medical Birth Registry of Norway 2006–2014.**

| **BMI Change**  **Units kg/m2** | **Pre-pregnant BMI <25 in second pregnancy** | | | | | |  |  | **Pre-pregnant BMI ≥ 25 in second pregnancy** | | | | |
| --- | --- | --- | --- | --- | --- | --- | --- | --- | --- | --- | --- | --- | --- |
|  | **N** | **GDM /1000** | **Crude RR** | **95% CI** | **a RR*** | **95% CI** |  | **N** | **GDM**  **/1000** | **Crude**  **RR** | **95% CI** | **a RR*** | **95% CI** |
| **<-2** | 7/1,091 | 6.4 | 1.0 | 0.5-2.2 | 1.1 | 0.5-2.5 |  | 8/601 | 13.3 | 0.5 | 0.2-0.96 | 0.5 | 0.2-1.2 |
| **-2 to < - 1** | 16/1,848 | 8.7 | 1.4 | 0.8-2.4 | 1.3 | 0.7-2.4 |  | 18/536 | 33.6 | 1.2 | 0.7-1.9 | 1.2 | 0.7-2.1 |
| **-1 to < 1** | 57/9,114 | 6.3 | 1.0 | Reference | 1.0 | Reference |  | 69/2,398 | 28.8 | 1.0 | Reference | 1.0 | Reference |
| **1 to <2** | 26/2,290 | 11.4 | 1.8 | 1.1-2.9 | 1.8 | 1.1-3.0 |  | 53/1,524 | 34.8 | 1.2 | 0.9-1.7 | 1.3 | 0.9-1.9 |
| **2 to <4** | 15/1,216 | 12.3 | 2.0 | 1.1-3.5 | 1.8 | 0.9-3.4 |  | 82/2,063 | 39.7 | 1.4 | 1.0-1.9 | 1.4 | 0.98-2.0 |
| **≥4** | 4/150 | 26.7 | 4.3 | 1.6-11.6 | 6.0 | 2.1-16.6 |  | 84/1,367 | 61.4 | 2.1 | 1.6-2.9 | 2.1 | 1.5-3.0 |
| **Total** | 125/15,709 | 8.0 | 15,709 |  | 13,526 |  |  | 314/8,489 | 37.0 | 8,489 |  | 7,298 |  |

*Adjusted (a) for maternal age in second pregnancy (<25 [reference], 25–29, 30–34, ≥35 years), maternal country of birth (Nordic [reference]/non-Nordic), maternal education (<11, 11–13, ≥14 [reference] years), smoking in pregnancy (no [reference],/yes), interpregnancy interval (<12, 12–23 [reference], 24–35, ≥36 months), and year of second birth (continuous).
