# Supplementary material for: Gestational diabetes mellitus and interpregnancy weight change: A population-based cohort study
Source: PLoS Med. 2017 Aug 1;14(8):e1002367. doi: 10.1371/journal.pmed.1002367 (PMC5538633; doi:10.1371/journal.pmed.1002367)
Supplement: S10 Table — *Mothers with first and second pregnancy between 2006–2014, without Gestational Diabetes Mellitus (GDM) in first pregnancy and without diabetes mellitus prior to first and second pregnancy. (DOCX) [file pmed.1002367.s013.docx]

|  | **Study population*** | | **Total population*** | |
| --- | --- | --- | --- | --- |
|  | ***n*** | **%** | ***n*** | **%** |
| **Gestational Diabetes Mellitus (GDM) in second pregnancy** |  |  |  |  |
| No | 23,759 | 98.2 | 78,015 | 98.4 |
| Yes | 439 | 1.8 | 1,269 | 1.6 |
| **Maternal age** (years) |  |  |  |  |
| <25 | 3,014 | 12.5 | 9,265 | 11.7 |
| 25-29 | 8,476 | 35.0 | 25,538 | 32.2 |
| 30-34 | 8,987 | 37.1 | 30,481 | 38.4 |
| ≥35 | 3,722 | 15.4 | 14,000 | 17.7 |
| **Maternal country of birth** |  |  |  |  |
| Nordic | 19,828 | 81.9 | 65,681 | 82.8 |
| Non-Nordic | 4,215 | 17.4 | 13,025 | 16.4 |
| Missing | 155 | 0.6 | 578 | 0.7 |
| **Maternal education** |  |  |  |  |
| <11 years | 3,460 | 14.3 | 10,569 | 13.3 |
| 11-13 years | 6,323 | 26.1 | 18,336 | 23.1 |
| ≥14 years | 13,678 | 56.5 | 48,125 | 60.7 |
| Missing | 737 | 3.0 | 2,254 | 2.8 |
| **Smoking** |  |  |  |  |
| No | 20,616 | 85.2 | 61,045 | 77.0 |
| Yes | 990 | 4.1 | 3,048 | 3.8 |
| Missing | 2,592 | 10.7 | 15,191 | 19.2 |
| **Inter-pregnancy interval** (months) |  |  |  |  |
| <12 | 4,806 | 19.9 | 13,814 | 17.5 |
| 12 to <24 | 10,342 | 42.7 | 31,874 | 40.4 |
| 24 to <36 | 5,811 | 24.0 | 19,714 | 25.0 |
| ≥36 | 3,210 | 13.3 | 13,498 | 17.1 |
| Missing | 29 | 0.1 | 384 | 0.5 |
| Total | 24,198 | 100 | 79,284 | 100 |

**S10 Table. The study population (*n* = 24,198) compared to the population with missing information on prepregnant Body Mass Index (BMI) in first and second pregnancy (*n* = 79,284).**

*Mothers with first and second pregnancy between 2006–2014, without GDM in first pregnancy and without diabetes mellitus prior to first and second pregnancy
